# Role of Zinc (Zn) in Human Reproduction: A Journey from Initial Spermatogenesis to Childbirth

**DOI:** 10.3390/ijms22042188

**Published:** 2021-02-22

**Authors:** Sundaram Vickram, Karunakaran Rohini, Subramanian Srinivasan, David Nancy Veenakumari, Kumar Archana, Krishnan Anbarasu, Palanivelu Jeyanthi, Sundaram Thanigaivel, Govindarajan Gulothungan, Nanmaran Rajendiran, Padmalayam Sadanandan Srikumar

**Affiliations:** 1 Department of Biotechnology, Saveetha School of Engineering, Saveetha Institute of Medical and Technical Sciences, Chennai, Tamil Nadu 602105, India; vickramas.16@gmail.com (S.V.); thanigaivel092@gmail.com (S.T.); 2 Unit of Biochemistry, Faculty of Medicine, AIMST University, Semeling, Bedong 08100, Kedah, Malaysia; 3 Department of Biomedical Engineering, Saveetha School of Engineering, Saveetha Institute of Medical and Technical Sciences, Chennai, Tamil Nadu 602105, India; srinivasans@gmail.com (S.S.); gulothungang@gmail.com (G.G.); nanmaran3263@gmail.com (N.R.); 4 BCX Bioorganics, Krishnasagara Village, Attibele, Bengaluru, Karnataka 562107, India; nancyveenakumari@gmail.com; 5 Department of Agriculture Engineering, Saveetha School of Engineering, Saveetha Institute of Medical and Technical Sciences, Chennai, Tamil Nadu 602105, India; archanatnau@gmail.com; 6 Department of Bioinformatics, Saveetha School of Engineering, Saveetha Institute of Medical and Technical Sciences, Chennai, Tamil Nadu 602105, India; anbu290785@gmail.com; 7 Department of Biotechnology, Vel Tech Rangarajan Dr. Sagunthala R&D Institute of Science and Technology, Chennai, Tamil Nadu 600062, India; jeypalanivelu91@gmail.com; 8 Unit of Psychiatry, Faculty of Medicine, AIMST University, Semeling, Bedong 08100, Malaysia; srikumar@aimst.edu.my

**Keywords:** zinc, seminal plasma, male infertility, human reproduction, cellular metabolism

## Abstract

Zinc (Zn), the second-most necessary trace element, is abundant in the human body. The human body lacks the capacity to store Zn; hence, the dietary intake of Zn is essential for various functions and metabolism. The uptake of Zn during its transport through the body is important for proper development of the three major accessory sex glands: the testis, epididymis, and prostate. It plays key roles in the initial stages of germ cell development and spermatogenesis, sperm cell development and maturation, ejaculation, liquefaction, the binding of spermatozoa and prostasomes, capacitation, and fertilization. The prostate releases more Zn into the seminal plasma during ejaculation, and it plays a significant role in sperm release and motility. During the maternal, labor, perinatal, and neonatal periods, the part of Zn is vital. The average dietary intake of Zn is in the range of 8–12 mg/day in developing countries during the maternal period. Globally, the dietary intake of Zn varies for pregnant and lactating mothers, but the average Zn intake is in the range of 9.6–11.2 mg/day. The absence of Zn and the consequences of this have been discussed using critical evidence. The events and functions of Zn related to successful fertilization have been summarized in detail. Briefly, our current review emphasizes the role of Zn at each stage of human reproduction, from the spermatogenesis process to childbirth. The role of Zn and its supplementation in in vitro fertilization (IVF) opens opportunities for future studies on reproductive biology.

## 1. Introduction

Zinc (Zn) is an essential trace element that is required for many normal bodily functions. Any malfunction or deficiency of zinc can cause abnormalities in the human body [1]. Zn deficiency is widespread in humans and common among various populations around the world [2]. Zn deficiency during the growth phase of humans results in growth failure due to hormonal imbalance and affects gonadal development and maturation [3]. The World Health Organization (WHO) has estimated that one-third of the world’s population is deficient in Zn [4], and this deficiency results in various human diseases. Zn is essential for both male and female reproductive potential as it is necessary for normal fertilization. It has been shown that the Zn content in semen is 85 to 90 times higher than in blood, thus protects the sperm cells from bacterial attack. Zn protects the sperm cells like a shield when entering the female reproductive tract and protects them from chromosomal damage [5]. There is a significant amount of evidence showing that Zn plays major contribution in initial spermatogenesis (from germ cells to sperm cells), sperm cell maturation in the epididymis, sperm cell motility, and pre-fertilization events in the female reproductive tract. It has its own functions during the pre-fertilization process, such as sperm capacitation, binding of the sperm zona pellucida (ZP), the acrosome reaction process, penetration at the site of the ZP, involvement in the sperm and oocyte binding process, egg or zygote activation, and the final zona reaction [6]. In addition, Zn plays important roles at various stages from post-fertilization until childbirth [7]. A low Zn concentration in the diet results in low sperm quality, as well as idiopathic male infertility. A reduction in Zn of less than 5–7 ppm results in the impairment of reproductive function in both males and females [8]. Zn plays an anti-inflammatory activity and possibly plays a protagonist in oxidation, as shown by various research findings [9]. The concentration of Zn in the testis increases during the early spermatogenesis period, which is shown by its regulatory activities on spermatogonial proliferation and its need in the maintenance of germ cells without any damage during the meiosis period [10]. Zn carries out regulatory activity through the transcription of thymidine kinase, an important enzyme for DNA synthesis [11]. Any alteration in thymidine kinase due to Zn deficiency will lead to impaired spermatogenesis and the arrest of germ cells. Zn plays an important action in testes development of an adult, allowing proper reproductive function [12]. Zn deficiency in the testes is positively correlated with hypogonadism, improper secondary sexual characteristics, and other reproductive issues [13]. The prostate releases more Zn into the seminal plasma during ejaculation and plays a significant action in sperm release and motility [14]. Decreased levels of Zn in seminal vesicle and prostate secretions will affect the coagulation properties of semen. Semen consistency is viscous in nature, and usually, hyperviscosity is related to seminal vesicle secretion [15]. Zn plays a major part in the production, storage, and transport of major sex hormones, especially testosterone [16]. Their transfer and fusion are mediated by the Zn ions present in both prostasomes and intracellular Zn ions. Zn deficiency is associated with an increased trend for cells and tissues to die on their own, a phenomenon known as apoptosis [17]. This has been proven to be a major cause of Zn-deficiency-related cell death. Zn supplementation restores the antioxidant capacity. Oral supplementation with Zn has been shown to work effectively for issues such as premature ejaculation and erectile dysfunction [18].

In this review, the critical roles of Zn at various stages, from testicular development to spermatogenesis, the conversion of germ cells into sperm cells, activities in accessory sex glands (including the prostate, epididymis, and seminal vesicles), sex hormones for monitoring various processes, ejaculation, in the female reproductive tract, in the pre-fertilization process (including capacitation), and from post-fertilization until childbirth are discussed. The significance of using oral Zn supplementation for advanced assisted reproduction technology is also covered.

## 2. Zinc (Zn) Trafficking in Sperm Transport

Zn has been very well elucidated in reproductive biology terms, but in terms of dynamic study at various stages of sperm production, the role of Zn has scarcely been studied and reviewed [19]. Zn trafficking through the membrane is endorsed by precise families of transporters known as the ZnTs, which are involved in effluent release [20]. Zn is involved in numerous sperm functions and is expected to exhibit maximal uptake during sperm transport after movement through the epididymis (post-epididymal phase) [21]. Zn release and uptake during male reproductive processes, from spermatogenesis to spermiation, are monitored and have been shown to be performed by the ZnTs [22]. From this critical point of view, the aforementioned study confirmed that there is a high Zn content in the testicular and epididymal phases, and significant changes (less content) occur in the ejaculation phase [23]. However, there is a lack of important evidence to prove that the gain in hypermotility (found through flow cytometry) may be the primary reason for the loss of Zn content during ejaculation. This study provided a scientific hypothesis suggesting that Zn supplementation could be used as a therapy for male infertility patients, but more research has to be performed in order to confirm the effects of Zn supplementation [24].

## 3. Role of Zn in Normal Spermatogenesis

**Figure 1:**
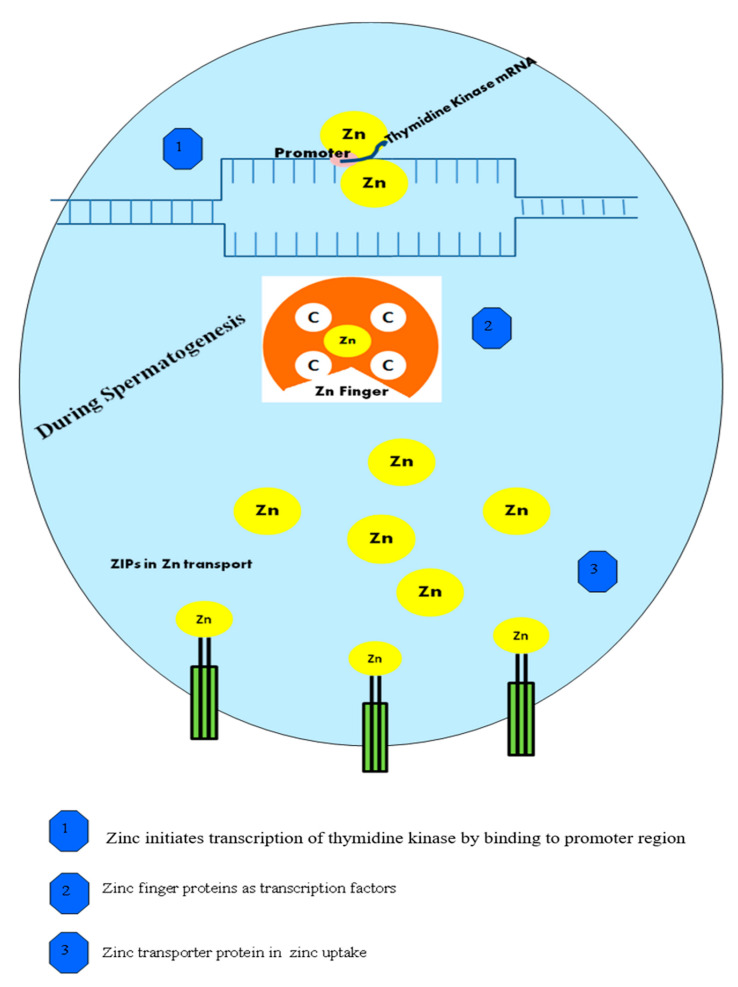
ZIPs (the zinc-regulated transporters, iron-regulated transporter-like proteins). The role of zinc (Zn) during spermatogenesis at the molecular level. The figure depicts how Zn initiates the transcription of kinase, Zn finger proteins as transcription factors, and the Zn uptake process during spermatogenesis.

The altered expression of Zn transporters will affect the Zn content, thus pave a way for poor spermatogenesis [25]. The need for Zn and its presence in germ cell survival before maturity, as well as in substitution by protamine during spermatogenesis, has been reported by Ellis in 2014 [26]. Zn plays a key parameter in spermatogenesis and in the early stage of sperm cell development because of its presence in the nucleus and chromatin and its accumulation in spermatocytes [27]. The concentration of Zn in the testes increases during the early spermatogenesis period, as evidenced by its regulatory activities in spermatogonial proliferation and during the meiosis period for the maintenance of germ cells without damage [28]. Zn reduction at this stage will result in unauthenticated proliferation of spermatogonia and to germ cell death without the formation of effective mature sperm cells [29]. This primes to a reduction in the number of mature sperm cells available in the testis in a batch; hence, it may incline to a reduction in the number of spermatozoa in the ejaculation fluid, which leads to the misdiagnosis of oligospermia [30]. Impaired spermatogenesis tends to impaired spermatozoa during ejaculation and a lower sperm count. The WHO stated that the sperm count should be around 20 million sperm per milliliter. A reduction in the count due to Zn deficiency during spermatogenesis [31] may help in diagnosis of oligospermia. There is strong evidence to support the importance of Zn during spermatogenesis and its implications on diagnosis [32]. A high Zn content in the prostate provides evidence of its critical act in epididymal transit, whereby Zn stabilizes the sperm cells during or before ejaculation [33]. The role of Zn in spermatogenesis is shown in Figure 1.

Zinc finger proteins (ZFPs) play a significant action in spermatogenesis [34]. Zn is a major component of ZFPs, a large class of transcription factors [35]. These transcription factors are essential and exhibit different functions during growth and development, including DNA binding, cell apoptosis, and activation of transcription and translational processes [36]. Many researchers have discussed the critical activity of ZFPs during the proliferation and differentiation of germ cells, as well as during spermatogenesis. ZFP185 plays a significant role in spermatogenesis through its overexpression in Leydig cells and leads to testosterone production [37].

Zinc transport proteins (ZIPs), which aid in Zn uptake into the cytoplasm and act at the intracellular zinc level, play a major part in spermatogenesis [38]. The reduced expression of ZIPs during the transport and uptake of Zn into the intracellular lumen may lead to impaired spermatogenesis at different stages [39]. A positive correlation exists between the Zn circulating level and the Zn intake level during spermatogenesis [40].

## 4. Action of Zn in the Testes Phase

The interaction of Zn with cadmium in the adult testes has been explored by many researchers [41]. Zn deficiency in the testes is positively correlated with hypogonadism and improper development of secondary sexual characteristics [42,43]. Zn and Cd interactions are due to similarities in ion pairs, and they form a competitive interaction [44]. Decreased Zn uptake by spermatogonia results in competitive substitution by cadmium, which results in a decreased Zn content and, in turn, the functions of sperm development in the testes are disturbed [45]. The reduced level of Zn in the testes leads to severe damage and reduced testes weight [40]. The germ cell capacity may be significantly reduced because of the abridged testes size due to Zn deficiency [46]. This will automatically lead to impaired spermatogenesis and will inhibit spermatid differentiation [47]. Zn deficiency in the testes also changes the structure of the Leydig cells and causes problems in proliferation and differentiation. Reduced levels of Zn in the testes and complications in the Leydig cells lead to reduced sex steroid levels, the impairment of spermatogenesis, and thus poor fertilization [48]. Reduced Zn in the testes also leads to oxidative damage to lipids, altered transcription and translation, and impaired DNA and proteins in testicular cells; hence, the quality of fertilization is poor [49]. In a rat model, cadmium-mediated toxicity and damage to the testes has been related to Zn competitive binding [50].

## 5. Significance of Zn in the Prostate

Zn is present throughout the human body. However, in the prostate, Zn is an essential substance, and it is present at high concentrations compared with other soft tissues [51,52]. The prostate releases more Zn into the seminal plasma during ejaculation, where it plays an important act in sperm release and motility [53]. The human prostate contains 150 μg/g of Zn in its tissues, which is three times higher than that in any other soft tissue. Similarly, prostatic fluid is also rich in Zn, with approximately 500 μg Zn/mL [54]. The major function of Zn in the prostate is to provide antimicrobial activity, which reduces sperm cell attack during ejaculation. The upper reproductive tract of females comprises a number of active microbes; once the sperm enters, it can be damaged by microbes [55]. Zn exhibits antimicrobial properties, allowing it to defend and protect the sperm cells from damage [56].

Zn plays a major part in the Krebs cycle and is utilized to ensure maximal production of citrate in prostatic fluid [57]. This is essential for the normal functioning of spermatozoa. Zn homeostasis is highly regulated in the prostate. Changes in Zn requirements may occur during sexual development. ZNT1 is needed for cellular proliferation, as evidenced by its reduced expression following the attainment of sexual maturity [58] and the accumulation of Zn in the prostate during this time [19].

## 6. The Mechanism of Action of Zn in Capacitation

Sperm capacitation is an important process in proper fertilization [59]. Zn spark is treated as a novel biomarker of the mammalian quality of embryos and other aspects of developmental potential. In terms of flux, little research has been done on Zn ions and their implications [60]. The proton extrusion mechanism plays a key parameter in capacitation. Numerous studies have shown its importance in voltage-gated proton channels [61]. This channel regulation is more important for the entry of Ca^2+^ ions through another channel called CatSper. This mechanism has been linked to the activation of protein tyrosine phosphorylation during capacitation [62]. Maintenance of the pH and proteasomal activities occur in the presence of Zn flux or spark [63].

## 7. Mechanism of Zn in Human Seminal Vesicles

In the later stages of ejaculation, human seminal vesicle secretion plays a significant role [64]. In proper fertilization, the five acts of seminal vesicles are: helping in semen coagulation, maintaining semen stability, enhancing sperm motility, inhibition of sperm motility, and different antioxidant functions [65]. Semen is viscous in nature. After ejaculation it comes into contact with the seminal vesicles and coagulates immediately [66]. Semenogelin proteins are a large part of the coagulum, and coagulation is mediated or activated by Zn ions [67]. For coagulation and inhibition of motility, these Zn ions are important. In seminal vesicle and prostate secretion, decreased levels of Zn can influence semen coagulation [68]. Semen consistency and seminal vesicle secretions are typically correlated with hyperviscosity [69]. Zn is a constituent of seminal vesicles, and hyper-viscosity arises when a high degree of secretion occurs. Hyperviscosity of semen is often associated with decreased motility, reduced normal morphology, and low volume of semen [70]. High chromatin stability occurs when a Zn chelating agent is present in abundance, which is attributed to high or hyperviscous semen samples [71]. Seminal vesicle hypofunction can contribute to semen sample hyperviscosity, and Zn plays a major role in this respect [72]. The seminal vesicles secrete prolactin, and it has been shown that it is associated with Zn. In sperm motility, prolactin plays a potential role and is considered to be a motility enhancer. This is one of the essential mechanisms associated with proper sperm motility following ejaculation [73].

## 8. Role of Zn in Major Sex Hormones

Zn plays a major role in the production, storage, and transport of major sex hormones, especially testosterone which is believed to be an important regulatory hormone for spermatogenesis [74,75]. Dietary-level Zn monitoring is important to determine the production of testosterone [76]. Therefore, during in vitro fertilization (IVF), Zn intake is monitored by experts. A deficiency in dietary Zn leads to an increase in circulating luteinizing hormone, but low levels of testosterone are found in the seminal plasma and serum [77]. This provides evidence of the important action of dietary Zn. Zn deficiency is positively correlated with a decreased or damaged population of Leydig cells, as well as with changes to proliferation and differentiation, Leydig cell apoptosis, and testes damage [78]. Zn deficiency leads to inflamed testes and oxidative damage to the Leydig cells [79]. Impaired spermatogenesis, a decreased testosterone concentration, damaged luteinizing hormone (LH) receptors, damaged Leydig cells, and a change in the appearance of the Leydig cells are common indicators of lower or deficient Zn levels [80].

## 9. Role of Zn in Prostasomes and Sperm-Binding Activity

Prostasomes are membranous extracellular vesicles found in the semen that are secreted by the prostate gland. Prostasomes are rich in lipids and phospholipid proteins [81,82]. Prostasomes and spermatozoa fusion process mediated by pH- and protein helps in proper fertilization. The amino peptidase present in prostasomes has to be transferred to the spermatozoa for proper motility [83]. The transfer and fusion process are mediated by Zn ions present in both prostasomes and intracellular Zn ions. Sperm has to acquire membrane-bound proteins, which is achieved through Zn ion-mediated transfer [84].

## 10. Role of Zn in Anti-Cell Death and Anti-Apoptosis

Evidence for the mechanism of action of Zn in apoptosis has been found in the last three decades of research [85]. Zn deficiency is associated with an increasing trend of cell and tissue death, a process known as apoptosis [17]. Germ cells must undergo several processes and reach the milestone of becoming sperm cells, the male gonads required for proper fertilization [86]. Zn deficiency in Leydig cells is associated with increased apoptosis and a change in the volume of the testes; this reduces the number of germ cells being converted into sperm cells [87]. Caspase 3 and Bcl-2 are important genes and proteins through which a Zn deficiency results in apoptosis [88]. The mechanism of Zn in providing protection from apoptosis is associated with many mechanisms. Further research is needed regarding the labile Zn that protects cells from damage, the mechanism through which the delivery of Zn to critical targets occurs. [89]. The regulation of apoptosis by Zn via Bcl 2 and caspase 3 plays a major part in cellular protection [90]. Many researchers have revealed the anti-apoptotic properties of Zn, but the mechanism by which Zn protects against apoptosis is not clearly understood, and it is different at different levels. DNA fragmentation may lead to a Ca^2+^- and Mg^2+^-dependent endonuclease action that results in apoptosis of the Leydig cells [91]. Zn has the capacity to inhibit these ions and thus prevent DNA fragmentation and apoptosis. Oxidative stress is another factor that affects the whole process and leads to apoptosis with increased levels of reactive oxygen species (ROS) in the serum and seminal plasma [92]. Zn acts as an antioxidant promoter and mediator that engulfs ROS through various means [93]. An increase in ROS is mediated by Zn deficiency and hence leads to oxidative stress-driven apoptosis [94]. Zn protects the sperm cell membranes by providing a coating layer through the mediation of SH (Sulfhydryl) group binding in proteins [95]. Sperm membrane fluidity increases and, hence, mediation of the proper fertilizing potential of spermatozoa occurs. In cases of Zn deficiency, there is an increased level of malondialdehyde in the serum and seminal plasma and reduced levels of antioxidants such as SoD (superoxide dismutase) [96].

## 11. Zn and Its Significance in Estrogen

Zn is an essential trace element in female reproductive physiology. In a study conducted using a rat model [97], Zn-deficient feed was administered to rats, resulting in reduced or inhibited concentrations of follicle-stimulating hormone and LH (luteinizing hormone) [98]. This result emphasizes the importance of Zn in female reproductive physiology. Zn deficiency also increases the occurrence of abnormal ovarian functions and disturbs the menstruation cycle, creating false hope of normal fertilization [99]. The mechanism or the basis of Zn in both male and female reproduction are based on interactions between Zn and hormone receptors [100]. In the absence of the Zn metalloenzyme, sex hormones in both male and female reproduction systems cannot be activated [101]. Zn metalloenzymes are bound to sex hormone receptors as a complex formation in the presence of RNA polymerase. Fetuin-A and B plays a major role in maintaining fertility status in female [102]. Gene knockout studies showed the importance of Fetuin-A in bone mineralization. Also, the animal model study proves the role of Fetuin B in female fertility status [102]. The absence or deficiency of this protein may cause female infertility due to zona pellucida hardening. This is caused by the presence of metalloproteinase ovastacin in non-fertilized oocytes [103]. Any malfunction or deficiency of Zn may lead to the prevention of binding of DNA and the hormone–receptor complex. This prevents the normal functions of estrogen from occurring [104]. Furthermore, the activators and regulatory potential of other genes presented here collapse, leading to failures in estrogen production and monitoring [105].

## 12. Zn as a Regulator in the Female Reproductive Tract

Once the sperm enter the female reproductive tract, numerous immune responses against sperm cell entry are activated [106]. The presence of Zn helps to reduce these responses and sends a signal that this is for reproductive action and that the process should not be disturbed [107]. This is because Zn acts as a cofactor for many proteins in the female reproductive tract and activates them, allowing complete fertilization competency [108]. Zn ions play a key parameter in sperm capacitation in the female reproductive tract and act as a regulating authority for other important events, ensuring effective fertilization [109]. Zn efflux is important for Ca^2+^ influx, and this process is mandatory for capacitation to occur. Any malfunction or deficiency in the process of zinc ion efflux will reduce capacitation [110].

**Figure 2:**
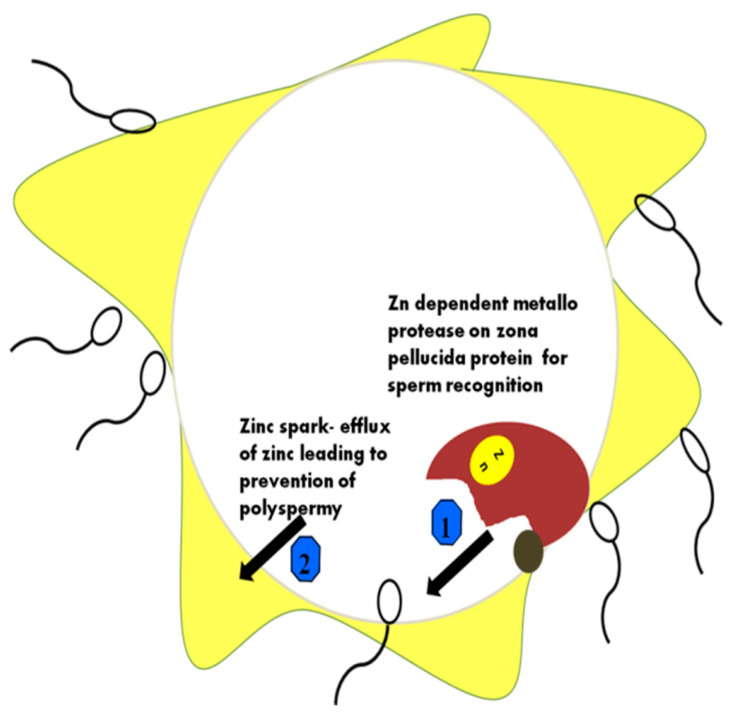
Different roles of Zn ions during sperm–ova interactions. Zona pellucida (ZP) hardening occurs through membrane protein changes when the ZP protein acts using the Zn-binding protease. The major functions of zinc in the prevention of polyspermy are depicted in the figure. 1—Zn dependent metalloprotease on zona pellucida for sperm recognition; 2—how Zn efflux results in polyspermy.

Anti-polyspermy is the need for effective sperm–oocyte interactions for embryo development. This prevents the entry of more than one sperm cell into the cytoplasm of an oocyte at the time of fertilization [111]. The anti-polyspermy defense mechanism is very complex, and a high level of understanding of the underlying mechanism is required. Embryo polyploidy lethality is the cause of polyspermy during fertilization. Two major mechanisms occur that act as a barrier to polyspermy action [112]. The first is membrane depolarization, and the second is cortical exocytosis. Zn^2+^ ions released from the cortex region of oocytes help to monitor these two mechanisms under which the polyspermy process is prevented [113]. This release is named Zn sparks, and its importance in the female reproductive tract has been shown by many scientists [114]. Zn regulates the entry of sperm into the oocytes using its anti-polyspermy capacity during this period for newly fertilized eggs. Zn could play a significance in the decapacitation of other sperm cells present near the fertilized egg [115]. This process is termed the zinc shield for the prevention of polyspermy-mediated pregnancy [116]. Other evidence shows that Zn^2+^ ions can inhibit the process of fertilization when added to IVF media components. Zn in the ZP mechanism is complicated, and the mechanism behind this process has not yet been elucidated [117]. The roles of Zn in various stages of sperm–ova interactions are shown in Figure 2.

## 13. Zn Supplementation for Male Fertility

Seminal fluid Zn^2+^ ions play a key parameter in boosting male fertility [118]. Any deficiency or lowering of the concentration of Zn^2+^ in the seminal plasma results in a low sperm count, as well as a low sperm quality [119]. Many researchers have shown a positive correlation between Zn ion concentration and sperm concentration, as well as the normal morphology of sperm cells. Zn supplementation in rats and uremic men results in increased sexual function and reduced sexual dysfunction [120]. Following supplementation of Zn with folate, an increase in the sperm count was observed in oligospermic patients [121]. By scavenging ROS in the semen and serum, Zn supplementation can restore the antioxidant capacity of Zn [122]. The supply of blood to the penile veins at the time of erection is monitored by a Zn-mediated process [123]. Oral supplementation of Zn was found to work effectively in cases of premature ejaculation and for patients with erectile dysfunction. Dietary Zn intake in fertility enhancement has not been scientifically proven; however, in dietary intake monitoring, the percentage of non-capacitated sperm cells was observed to increase during ejaculation [124]. The mechanism responsible for this process has not been elucidated in a scientific manner. Although many researchers have identified the effects of oral Zn supplementation on both male and female reproductive functions and used Zn therapy as a measure for increasing sexual function, there is also evidence to show that Zn intake higher than 100 mg/day is associated with prostate cancer [125]. There is no proof that Zn acts as a carcinogen [126], but researchers have claimed that there should be a limit on Zn intake, as a sudden increase in Zn intake could lead to prostate cancer [54]. Zn intake up to 100 mg/day is not associated with risk of prostate cancer, but supplementing Zn more than 100 mg/day may lead to prostate cancer risk [54].

## 14. Roles of Zn in Maternal, Perinatal, and Postnatal Healthcare

Globally, decreasing the mortality rates of perinatal, neonatal, and early childhood infants is the biggest challenge for researchers and clinicians [127]. Proper monitoring of micro- and macronutrient intakes and supplementation for women can reduce the mortality rate [128]. This process is performed more extensively in developing countries than in developed countries. Zn plays a major action in maternal, infant, and neonatal survival [129]. The importance of Zn during the maternal period and birth has not been well elucidated [130]. The average dietary Zn intake lies in the range 8–12 mg Zn/day in developing countries during the maternal period. Worldwide, the dietary Zn intake varies for pregnant and lactating mothers, but the average intake lies between 9.6 and 11.2 mg Zn/day [131]. Zn intake also slightly increases by drinking water, but the intake should be a minimum of 4 L/d [132]. An insignificant Zn deficiency during maternity is associated with a lower birthweight, and a high Zn deficiency can lead to spontaneous abortion and various abnormalities, especially congenital ones [133]. Mild Zn deficiency in pregnant women can lead to many complications in stages 1 and 2 of labor, such as premature rupture of the membranes, which sometimes necessitates the use of operative measures in childbirth [134]. Oxytocin secretion can be monitored using Zn, and it acts as a cofactor during this time [135]. These complications further result in neonatal sepsis, neonatal asphyxia, and respiratory distress [136]. Zn deficiency in the mother can be inherited by the infant. These infants may display symptoms such as alopecia, appetite loss, diarrhea, impaired immune related functions, and dermatitis [137]. This type of Zn deficiency disorder found in premature babies and infants occurs because of a lack of zinc in breast milk. Pedigree analysis has shown the inheritance nature of zinc deficiency from the mother (Zn-deficient breast milk) to babies. Also, it was found that maternal Zn supplementation did not increase the Zn level in breast milk [137]. The important studies on Zn and reproduction in recent years are summarized in Table 1.

## 15. Conclusions

The dietary intake of Zn plays an essential role in the reproductive potential of both males and females. The human body cannot store Zn, so dietary consumption is the only way to maintain the body’s metabolism, especially for men and women of reproductive age. The WHO has reported on the global diseases or syndromes related to Zn deficiency. Women need a higher dietary Zn intake than men at reproductive age and during the maternity period. Zinc transporters are prevalent throughout the genital tract, so Zn is taken up throughout the process of sperm development, from germ cells to mature sperm cells. During spermatogenesis, a small amount of Zn is sufficient, but the need for Zn increases when maturation is reached; that is, at the time of epididymal transit. After this process, prostate Zn secretions will overcome spermatozoa and act as a defense at the time of ejaculation. When the female reproductive tract is reached, both seminal Zn and female Zn contents in the tract have a combined effect on ensuring a clean path to fertilization. Seminal Zn acts as a cofactor for the semenogelin protein and helps with gel formation or liquefaction. Zn helps in motility, especially forward-directional motility, with the help of fusion of prostasomes into spermatozoa membranes and the transfer of all essential components. Zn facilitates capacitation and ZP binding via multiple mechanisms. In the upper reproductive tract secretions, Zn intermediates pre-fertilization process, but the mechanism through which this occurs is not well understood yet. Zn plays a key role in the penetration of sperm into oocytes to form a mature zygote, as well as in the post-fertilization period. Zn supplementation during pregnancy and the perinatal and neonatal periods has been well discussed. Overall, Zn supplementation leads to successful outcomes in more than 50% of infertile cases. Zn supplementation is essential for males and females undergoing infertility treatment. A positive correlation with pregnancy outcomes exists for ART (assisted reproductive technology) methods that involve Zn supplementation as a part of the treatment. Although the mechanism behind this has not been elucidated, many ART centers prefer to use Zn supplementation. In this review, we have summarized the major functions and mechanism of Zn and the need for this element from spermatogenesis to postnatal care.

## Figures and Tables

**Table 1:** Important investigations into the roles of Zn in human reproduction and human male infertility and its implications conducted in the last 13 years.

Author and Year	Zn Role in Human Reproduction and Infertility	Study Conclusion
Qu et al., 2007 [138]	Zn-α2-glycoprotein, termed ZAG, plays a major action in sperm motility.	ZAG could be present in human semen, and it could help with proper motility as well as with the signaling pathway known as PKA (Protein Kinase A).
Saleh, 2008 [139]	Semen contains higher concentrations of Zn and copper than any other body fluid. This helps to maintain sperm quality.	For proper diagnosis of male infertility, Zn and Cu estimation is important.
Colagar et al., 2009 [140]	The absence or moderate deficiency of Zn in the seminal plasma leads to increased reactive oxygen species (ROS) and increased oxidative damage, which could result in low sperm quality.	The seminal Zn concentration was found to be significantly positively correlated with sperm count and the normal morphology of sperm. A low or absent Zn intake results in low semen quality and leads to idiopathic male infertility.
Dissanayake et al., 2010 [141]	Zn plays major parameter in determining sperm count, normal sperm morphology, and other parameters.	Both the Zn concentration and total amount of Zn per volume of ejaculate were calculated in this study. The total Zn content was termed Zn (T), and it was positively correlated with the sperm count and normal morphology.
Khan et al., 2011 [142]	Zn deficiency plays a key act in human male infertility. Zn deficiency is associated with hypogonadism and deficient development of secondary sex characteristics.	Having adequate Zn in the seminal plasma aids in proper sperm functioning. An increased amount of Zn results in decreased sperm motility, but a decreased amount of Zn in the seminal plasma was associated with an increased sperm count. It is very crucial to monitor the Zn content in seminal plasma.
Hadwan et al., 2012 [143]	Human seminal Zn is classified into three types of ligands: high, intermediate, and low molecular weight ligands. An increase in the oral supplementation of Zn results in increased sperm motility for asthenospermic patients.	This study concludes that the overall increase in motility in asthenospermic patients following Zn supplementation increases the overall high and low molecular Zn ligand levels.
Sundaram et al., 2013 [144]	Zn acts as a cofactor for DNA binding proteins and Zn fingers.	Zn could be the best biochemical marker for major semen anomalies, as well as for the proper diagnosis of human male infertility.
Foresta, 2014 [20]	Zn is involved in a number of sperm functions after the post-epididymal phase reaches a maximum.	During the entire lifetime of sperm, Zn trafficking occurs.
Altaher and Abdrabo, 2015 [145]	Zn and Cu play major actions in oligospermic and asthenospermic patients.	Zn concentration was significantly lower in cases of azoospermia and oligospermia.
Zhao et al., 2016 [146]	Systematic data analysis suggests that Zn concentration is significantly lower than in other fertile groups, which proves the significance of Zn in semen parameters.	Zn supplementation increases the major semen parameters like semen volume, forwarded motility, and normal morphology.
Nenkova et al., 2017 [137]	Zn plays a major action in protecting spermatozoa against oxidative stress.	There is evidence that trace elements play an antioxidative role at the time of ejaculation.
Fallah et al., 2018 [1]	Zn acts as an antibacterial agent in the female genital tract and even helps in protection from immunological shock.	Zn could be considered as a nutrient marker for male reproductive potential.
Mirnamniha et al., 2019 [6]	Zn plays a major function in the incitation of capacitation.	The measurement of Zn in the seminal plasma of idiopathic male infertility is essential.
Vickram et al., 2020 [84]	Zn plays a major function in mediating the binding of prostasomes on spermatozoa to transfer essential compounds, which paves the way for fertilization.	Prostasomes are biomarkers for both male infertility and prostate cancer diagnosis.

## References

[1] Fallah A., Mohammad-Hasani A., Colagar A.H. (2018). Zinc is an essential element for male fertility: A review of Zn roles in men’s health, germination, sperm quality, and fertilization. J. Reprod. Infertil..

[2] Shukla A.K., Tiwari P.K., Pakhare A., Prakash C. (2016). Zinc and iron in soil, plant, animal and human health. Indian J. Fertil..

[3] Prasad A.S. (2017). Trace metals in growth and sexual maturation. Metabolism of Trace Metals in Man Volume I (1984): Developmental Aspects.

[4] World Health Organization (2019). Global Status Report on Alcohol and Health 2018.

[5] Kovacik A., Tirpak F., Tomka M., Miskeje M., Tvrda E., Arvay J., Fik M. (2018). Trace elements content in semen and their interactions with sperm quality and RedOx status in freshwater fish Cyprinus carpio: A correlation study. J. Trace Elem. Med. Biol..

[6] Mirnamniha M., Faroughi F., Tahmasbpour E., Ebrahimi P., Harchegani A.B. (2019). An overview on role of some trace elements in human reproductive health, sperm function and fertilization process. Rev. Environ. Health.

[7] Wessels I., Maywald M., Rink L. (2017). Zinc as a gatekeeper of immune function. Nutrients.

[8] Nadjarzadeh A., Mehrsai A., Mostafavi E., Gohari M.R., Shidfar F. (2013). The association between dietary antioxidant intake and semen quality in infertile men. Med. J. Islamic Repub. Iran.

[9] Choi S., Liu X., Pan Z. (2018). Zinc deficiency and cellular oxidative stress: Prognostic implications in cardiovascular diseases. Acta Pharmacol. Sin..

[10] Kaur G., Thompson L.A., Dufour J.M. (2014). Sertoli cells–immunological sentinels of spermatogenesis. Semin. Cell Dev. Biol..

[11] Baltaci A.K., Mogulkoc R., Baltaci S.B. (2019). The role of zinc in the endocrine system. Pak. J. Pharm. Sci..

[12] Gandhi J., Hernandez R.J., Chen A., Smith N.L., Sheynkin Y.R., Joshi G., Khan S.A. (2017). Impaired hypothalamic-pituitary-testicular axis activity, spermatogenesis, and sperm function promote infertility in males with lead poisoning. Zygote.

[13] Prasad A.S. (2013). Discovery of human zinc deficiency: Its impact on human health and disease. Adv. Nutr..

[14] Samanta L., Parida R., Dias T.R., Agarwal A. (2018). The enigmatic seminal plasma: A proteomics insight from ejaculation to fertilization. Reprod. Biol. Endocrinol..

[15] Brazdova A. (2014). Study of Immunological Properties of Sperm and Seminal Plasma Antigens: Anti-Seminal and Anti-Sperm Antibodies in Female Immune Infertility: Characterization of Targeted Proteins. Doctoral dissertation.

[16] Egwurugwu J.N., Ifedi C.U., Uchefuna R.C., Ezeokafor E.N., Alagwu E.A. (2013). Effects of zinc on male sex hormones and semen quality in rats. Niger. J. Physiol. Sci..

[17] Roscioli E., Hamon R., Lester S., Murgia C., Grant J., Zalewski P. (2013). Zinc-rich inhibitor of apoptosis proteins (IAPs) as regulatory factors in the epithelium of normal and inflamed airways. Biometals.

[18] Hadwan M.H., Almashhedy L.A., Alsalman A.S. (2013). The key role of zinc in enhancement of total antioxidant levels in spermatozoa of patients with asthenozoospermia. Am. J. Respir. Cell Mol. Biol..

[19] Kambe T., Tsuji T., Hashimoto A., Itsumura N. (2015). The physiological, biochemical, and molecular roles of zinc transporters in zinc homeostasis and metabolism. Physiol. Rev..

[20] Foresta C., Garolla A., Cosci I., Menegazzo M., Ferigo M., Gandin V., De Toni L. (2014). Role of zinc trafficking in male fertility: From germ to sperm. Hum. Reprod..

[21] Babaei H., Abshenas J. (2013). Zinc therapy improves adverse effects of long term administration of copper on epididymal sperm quality of rats. Iran. J. Reprod. Med..

[22] Kambe T., Hashimoto A., Fujimoto S. (2014). Current understanding of ZIP and ZnT zinc transporters in human health and diseases. Cell. Mol. Life Sci..

[23] Vickram A.S., Das R., Srinivas M.S., Rao K.A., Jayaraman G., Sridharan T.B. (2013). Prediction of Zn concentration in human seminal plasma of Normospermia samples by Artificial Neural Networks (ANN). J. Assist. Reprod. Genet..

[24] Kerns K., Zigo M., Drobnis E.Z., Sutovsky M., Sutovsky P. (2013). Zinc ion flux during mammalian sperm capacitation. Nat. Commun..

[25] Kong B.Y., Duncan F.E., Que E.L., Kim A.M., O’Halloran T.V., Woodruff T.K. (2014). Maternally-derived zinc transporters ZIP6 and ZIP10 drive the mammalian oocyte-to-egg transition. Mol. Hum. Reprod..

[26] Ellis R.E., Stanfield G.M. (2014). The regulation of spermatogenesis and sperm function in nematodes. Semin. Cell Dev. Biol..

[27] Zhao Y., Zhao H., Zhai X., Dai J., Jiang X., Wang G., Li W., Cai L. (2013). Effects of Zn deficiency, antioxidants, and low-dose radiation on diabetic oxidative damage and cell death in the testis. Toxicol. Mech. Methods.

[28] Hai Y., Hou J., Liu Y., Liu Y., Yang H., Li Z., He Z. (2014). The roles and regulation of Sertoli cells in fate determinations of spermatogonial stem cells and spermatogenesis. Semin. Cell Dev. Biol..

[29] Bradbury N.A. (2017). All cells have a sex: Studies of sex chromosome function at the cellular level. Principles of Gender-Specific Medicine.

[30] Olesen I.A., Joensen U.N., Petersen J.H., Almstrup K., Rajpert-De Meyts E., Carlsen E., McLachlan R., Juul A., Jørgensen N. (2018). Decrease in semen quality and Leydig cell function in infertile men: A longitudinal study. Hum. Reprod..

[31] Omu A.E., Al-Azemi M.K., Al-Maghrebi M., Mathew C.T., Omu F.E., Kehinde E.O., Anim J.T., Oriowo M.A., Memon A. (2015). Molecular basis for the effects of zinc deficiency on spermatogenesis: An experimental study in the Sprague-dawley rat model. Indian J. Urol. IJU: J. Urol. Soc. India.

[32] Walczak–Jedrzejowska R., Wolski J.K., Slowikowska–Hilczer J. (2013). The role of oxidative stress and antioxidants in male fertility. Cent. Eur. J. Urol..

[33] Bolanca I., Obhodas J., Ljiljak D., Matjacic L., Kuna K. (2016). Synergetic effects of K, Ca, Cu and Zn in human semen in relation to parameters indicative of spontaneous hyperactivation of spermatozoa. PLoS ONE.

[34] Ishizuka M., Ohtsuka E., Inoue A., Odaka M., Ohshima H., Tamura N., Yoshida K., Sako N., Baba T., Kashiwabara S. (2016). Abnormal spermatogenesis and male infertility in testicular zinc finger protein Zfp318-knockout mice. Dev. Growth Differ..

[35] Ecco G., Imbeault M., Trono D. (2017). KRAB zinc finger proteins. Development.

[36] Lim K.H., Park S.G. (2015). Transcriptional regulation of KRAB-ZFPs in cancer. Mol. Cell. Toxicol..

[37] Harchegani A.B., Dahan H., Tahmasbpour E., Shahriary A. (2020). Effects of zinc deficiency on impaired spermatogenesis and male infertility: The role of oxidative stress, inflammation and apoptosis. Hum. Fertil..

[38] Thomas P., Converse A., Berg H.A. (2018). ZIP9, a novel membrane androgen receptor and zinc transporter protein. Gen. Comp. Endocrinol..

[39] Karweina D., Kreuzer-Redmer S., Müller U., Franken T., Pieper R., Baron U., Olek S., Zentek J., Brockmann G.A. (2015). The zinc concentration in the diet and the length of the feeding period affect the methylation status of the ZIP4 zinc transporter gene in piglets. PLoS ONE.

[40] Anjum M.R., Madhu P., Reddy K.P., Reddy P.S. (2017). The protective effects of zinc in lead-induced testicular and epididymal toxicity in Wistar rats. Toxicol. Ind. Health.

[41] Chemek M., Mimouna S.B., Boughammoura S., Delbès G., Messaoudi I. (2016). Protective role of zinc against the toxicity induced by exposure to cadmium during gestation and lactation on testis development. Reprod. Toxicol..

[42] Torabi F., Shafaroudi M.M., Rezaei N. (2017). Combined protective effect of zinc oxide nanoparticles and melatonin on cyclophosphamide-induced toxicity in testicular histology and sperm parameters in adult Wistar rats. Int. J. Reprod. Biomed..

[43] Tirabassi G., Biagioli A., Balercia G. (2014). Bone benefits of testosterone replacement therapy in male hypogonadism. Panminerva Med..

[44] Sarwar N., Ishaq W., Farid G., Shaheen M.R., Imran M., Geng M., Hussain S. (2015). Zinc–cadmium interactions: Impact on wheat physiology and mineral acquisition. Ecotoxicol. Environ. Saf..

[45] Bayer A.R. (2018). Zinc Dynamics during Murine Gamete and Embryo Development. Ph.D. Thesis.

[46] Lee Y.A., Kim Y.H., Ha S.J., Kim K.J., Kim B.J., Kim B.G., Choi S.-H., Kim I.-C., Schmidt J.A., Ryu B.Y. (2014). Cryopreservation of porcine spermatogonial stem cells by slow-freezing testis tissue in trehalose. J. Anim. Sci..

[47] Jordan M.V.C., Lo S.T., Chen S., Preihs C., Chirayil S., Zhang S., Kapur P., Li W.-H., De Leon-Rodriguez L.M., Lubag A.J.M. (2016). Zinc-sensitive MRI contrast agent detects differential release of Zn (II) ions from the healthy vs. malignant mouse prostate. Proc. Natl. Acad. Sci. USA.

[48] Niwas Jangir R., Chand Jain G. (2014). Diabetes mellitus induced impairment of male reproductive functions: A review. Curr. Diabetes Rev..

[49] Bisht S., Faiq M., Tolahunase M., Dada R. (2017). Oxidative stress and male infertility. Nat. Rev. Urol..

[50] Thévenod F., Lee W.K. (2013). Toxicology of cadmium and its damage to mammalian organs. Cadmium: From Toxicity to Essentiality.

[51] Verze P., Cai T., Lorenzetti S. (2016). The role of the prostate in male fertility, health and disease. Nat. Rev. Urol..

[52] Prashanth L., Kattapagari K.K., Chitturi R.T., Baddam V.R.R., Prasad L.K. (2015). A review on role of essential trace elements in health and disease. J. Dr. NTR Univ. Health Sci..

[53] Agarwal A., Durairajanayagam D., Halabi J., Peng J., Vazquez-Levin M. (2014). Proteomics, oxidative stress and male infertility. Reprod. Biomed. Online.

[54] Leitzmann M.F., Stampfer M.J., Wu K., Colditz G.A., Willett W.C., Giovannucci E.L. (2003). Zinc supplement use and risk of prostate cancer. J. Natl. Cancer Inst..

[55] Rametse C.L., Olivier A.J., Masson L., Barnabas S., McKinnon L.R., Ngcapu S., Liebenberg L.J., Jaumdally S.Z., Gray C.M., Jaspan H.B. (2014). Role of semen in altering the balance between inflammation and tolerance in the female genital tract: Does it contribute to HIV risk?. Viral Immunol..

[56] Jeng H.A., Huang Y.L., Pan C.H., Diawara N. (2015). Role of low exposure to metals as male reproductive toxicants. Int. J. Environ. Health Res..

[57] Franz M.C., Anderle P., Bürzle M., Suzuki Y., Freeman M.R., Hediger M.A., Kovacs G. (2013). Zinc transporters in prostate cancer. Mol. Asp. Med..

[58] Shusterman E., Beharier O., Shiri L., Zarivach R., Etzion Y., Campbell C.R., Lee I.-H., Okabayashi K., Dinudom A., Cook D.I. (2014). ZnT-1 extrudes zinc from mammalian cells functioning as a Zn^2+^ /H^+^ exchanger. Metallomics.

[59] Gangwar D.K., Atreja S.K. (2015). Signalling events and associated pathways related to the mammalian sperm capacitation. Reprod. Domest. Anim..

[60] Mendoza A.D., Woodruff T.K., Wignall S.M., O’Halloran T.V. (2017). Zinc availability during germline development impacts embryo viability in Caenorhabditis elegans. Comp. Biochem. Physiol. Part C Toxicol. Pharmacol..

[61] Seredenina T., Demaurex N., Krause K.H. (2015). Voltage-gated proton channels as novel drug targets: From NADPH oxidase regulation to sperm biology. Antioxid. Redox Signal..

[62] González-Fernández L., Macías-García B., Loux S.C., Varner D.D., Hinrichs K. (2013). Focal adhesion kinases and calcium/calmodulin-dependent protein kinases regulate protein tyrosine phosphorylation in stallion sperm. Biol. Reprod..

[63] Caldeira M.V., Salazar I.L., Curcio M., Canzoniero L.M., Duarte C.B. (2014). Role of the ubiquitin–proteasome system in brain ischemia: Friend or foe?. Prog. Neurobiol..

[64] La Vignera S., Condorelli R.A., Vicari E., Lotti F., Favilla V., Morgia G., Calogero A.E. (2013). Seminal vesicles and diabetic neuropathy: Ultrasound evaluation after prolonged treatment with a selective phosphodiesterase-5 inhibitor. Andrology.

[65] Stasinou T., Bourdoumis A., Owegie P., Kachrilas S., Buchholz N., Masood J. (2015). Calcification of the vas deferens and seminal vesicles: A review. Can. J. Urol..

[66] Puppo V., Puppo G. (2016). Comprehensive review of the anatomy and physiology of male ejaculation: Premature ejaculation is not a disease. Clin. Anat..

[67] Roan N.R., Liu H., Usmani S.M., Neidleman J., Müller J.A., Avila-Herrera A., Gawanbacht A., Zirafi O., Chu S., Dong M. (2014). Liquefaction of semen generates and later degrades a conserved semenogelin peptide that enhances HIV infection. J. Virol..

[68] Du Plessis S.S., Gokul S., Agarwal A. (2013). Semen hyperviscosity: Causes, consequences, and cures. Front. Biosci..

[69] Silverberg K.M., Turner T. (2017). Evaluation of sperm. Textbook of Assisted Reproductive Techniques: Volume 1: Laboratory Perspectives.

[70] Hamad A.W.R., Al-Daghistani H.I., Shquirat W.D., Abdel-Dayem M., Al-Swaifi M. (2014). Sodium, potassium, calcium and copper levels in seminal plasma are associated with sperm quality in fertile and infertile men. Biochem Pharm..

[71] Boshoff N.H. (2014). The Influence of Genotype on Sperm Motility and Sperm Head Morphometry of Merino (*Ovis*
*aries*) Sheep. Ph.D. Thesis.

[72] Barak S., Baker H.W.G., Feingold K.R., Anawalt B., Boyce A., Chrousos G., de Herder W.W., Dungan K., Grossman A., Hershman J.M., Hofland J., Kaltsas G. (2016). Clinical Management of Male Infertility.

[73] Marques P.I.F. (2016). An Evolutionary Perspective into the Role Of Kallikreins (KLKs) in Male Reproductive Biology.

[74] Peterson M.P., Rosvall K.A., Taylor C.A., Lopez J.A., Choi J.H., Ziegenfus C., Tang H., Colbourne J.K., Ketterson E.D., Tang H. (2014). Potential for sexual conflict assessed via testosterone-mediated transcriptional changes in liver and muscle of a songbird. J. Exp. Biol..

[75] Teerds K.J., Huhtaniemi I.T. (2015). Morphological and functional maturation of Leydig cells: From rodent models to primates. Hum. Reprod. Update.

[76] Chu Q., Chi Z.H., Zhang X., Liang D., Wang X., Zhao Y., Zhang L., Zhang P. (2016). A potential role for zinc transporter 7 in testosterone synthesis in mouse Leydig tumor cells. Int. J. Mol. Med..

[77] Sengupta P., Dutta S. (2018). Thyroid disorders and semen quality. Biomed. Pharmacol. J..

[78] Maremanda K.P., Khan S., Jena G. (2014). Zinc protects cyclophosphamide-induced testicular damage in rat: Involvement of metallothionein, tesmin and Nrf2. Biochem. Biophys. Res. Commun..

[79] Acharyya S. (2020). Inflammation and Ageing: Probable role in Male infertility. Chem. Biol. Lett..

[80] Carruthers M. (2013). Testosterone deficiency syndrome: Cellular and molecular mechanism of action. Curr. Aging Sci..

[81] Drabovich A.P., Saraon P., Jarvi K., Diamandis E.P. (2014). Seminal plasma as a diagnostic fluid for male reproductive system disorders. Nat. Rev. Urol..

[82] Aalberts M., Stout T.A., Stoorvogel W. (2014). Prostasomes: Extracellular vesicles from the prostate. Reproduction.

[83] Aalberts M., Sostaric E., Wubbolts R., Wauben M.W., Nolte E.N., Gadella B.M., Stout T.A., Stoorvogel W. (2013). Spermatozoa recruit prostasomes in response to capacitation induction. Biochim. Biophys. Acta (BBA) Proteins Proteom..

[84] Vickram A.S., Samad H.A., Latheef S.K., Chakraborty S., Dhama K., Sridharan T.B., Sundaram T., Gulothungan G. (2020). Human prostasomes an extracellular vesicle–Biomarkers for male infertility and prostrate cancer: The journey from identification to current knowledge. Int. J. Biol. Macromol..

[85] Liu Y., Batchuluun B., Ho L., Zhu D., Prentice K.J., Bhattacharjee A., Pourasgari F., Hardy A.B., Taylor K.M., Gaisano H. (2015). Characterization of Zinc Influx Transporters (ZIPs) in Pancreatic β Cells Roles in Regulating Cytosolic Zinc Homeostasis and Insulin Secretion. J. Biol. Chem..

[86] Goossens E., Van Saen D., Tournaye H. (2013). Spermatogonial stem cell preservation and transplantation: From research to clinic. Hum. Reprod..

[87] Asadi N., Bahmani M., Kheradmand A., Rafieian-Kopaei M. (2017). The impact of oxidative stress on testicular function and the role of antioxidants in improving it: A review. J. Clin. Diagn. Res..

[88] Thomas S., Quinn B.A., Das S.K., Dash R., Emdad L., Dasgupta S., Wang X.-Y., Dent P., Reed J.C., Pellecchia M. (2013). Targeting the Bcl-2 family for cancer therapy. Expert Opin. Ther. Targets.

[89] Zhang X., Liang D., Guo B., Deng W., Chi Z.H., Cai Y., Wang L., Ma J. (2013). Zinc transporter 5 and zinc transporter 7 induced by high glucose protects peritoneal mesothelial cells from undergoing apoptosis. Cell. Signal..

[90] Siddiqui W.A., Ahad A., Ahsan H. (2015). The mystery of BCL2 family: Bcl-2 proteins and apoptosis: An update. Arch. Toxicol..

[91] Evgeni E., Charalabopoulos K., Asimakopoulos B. (2014). Human sperm DNA fragmentation and its correlation with conventional semen parameters. J. Reprod. Infertil..

[92] Darbandi M., Darbandi S., Agarwal A., Sengupta P., Durairajanayagam D., Henkel R., Sadeghi M.R. (2018). Reactive oxygen species and male reproductive hormones. Reprod. Biol. Endocrinol..

[93] Dunnill C., Patton T., Brennan J., Barrett J., Dryden M., Cooke J., Leaper D., Georgopoulos N.T. (2017). Reactive oxygen species (ROS) and wound healing: The functional role of ROS and emerging ROS-modulating technologies for augmentation of the healing process. Int. Wound J..

[94] McCord M.C., Aizenman E. (2014). The role of intracellular zinc release in aging, oxidative stress, and Alzheimer’s disease. Front. Aging Neurosci..

[95] Li Z., Li Y., Zhou X., Cao Y., Li C. (2017). Preventive effects of supplemental dietary zinc on heat-induced damage in the epididymis of boars. J. Therm. Biol..

[96] Agarwal A., Virk G., Ong C., Du Plessis S.S. (2014). Effect of oxidative stress on male reproduction. World J. Men’s Health.

[97] Kirsten T.B., Queiroz-Hazarbassanov N., Bernardi M.M., Felicio L.F. (2015). Prenatal zinc prevents communication impairments and BDNF disturbance in a rat model of autism induced by prenatal lipopolysaccharide exposure. Life Sci..

[98] Barsony J., Manigrasso M.B., Xu Q., Tam H., Verbalis J.G. (2013). Chronic hyponatremia exacerbates multiple manifestations of senescence in male rats. Age.

[99] Garcia Barros R., Franciosi F., Dall Acqua P., Dieci C., Lodde V., Luciano A. (2019). Zinc supplementation during in vitro culture of bovine growing oocytes. Proceedings of the Technologies and controversies in reproduction. International conference proceedings.

[100] Hojyo S., Fukada T. (2016). Roles of zinc signaling in the immune system. J. Immunol. Res..

[101] Prasad A.S. (2013). Biochemistry of Zinc.

[102] Dietzel E., Wessling J., Floehr J., Schäfer C., Ensslen S., Denecke B., Rösing B., Neulen J., Veitinger T., Spehr M. (2013). Fetuin-B, a liver-derived plasma protein is essential for fertilization. Dev. Cell.

[103] Karmilin K., Schmitz C., Kuske M., Körschgen H., Olf M., Meyer K., Hildebrand A., Felten M., Fridrich S., Yiallouros I. (2019). Mammalian plasma fetuin-B is a selective inhibitor of ovastacin and meprin metalloproteinases. Sci. Rep..

[104] Roy B., Baghel R.P.S., Mohanty T.K., Mondal G. (2013). Zinc and male reproduction in domestic animals: A Review. Indian J. Anim. Nutr..

[105] Liang J., Shang Y. (2013). Estrogen and cancer. Annu. Rev. Physiol..

[106] Holt W.V., Fazeli A. (2015). Do sperm possess a molecular passport? Mechanistic insights into sperm selection in the female reproductive tract. MHR: Basic Sci. Reprod. Med..

[107] Pfaus J.G., Jones S.L., Flanagan-Cato L.M., Blaustein J.D. (2015). Female sexual behavior. Physiology of Reproduction.

[108] Yeste M., Jones C., Amdani S.N., Patel S., Coward K. (2016). Oocyte activation deficiency: A role for an oocyte contribution?. Hum. Reprod. Update.

[109] De Jonge C. (2017). Biological basis for human capacitation—revisited. Hum. Reprod. Update.

[110] Que E.L., Bleher R., Duncan F.E., Kong B.Y., Gleber S.C., Vogt S., Chen S., Garwin S.A., Bayer A.R., Dravid V.P. (2019). Quantitative mapping of zinc fluxes in the mammalian egg reveals the origin of fertilization-induced zinc sparks. Nat. Chem..

[111] Yi Y.J., Sutovsky M., Song W.H., Sutovsky P. (2015). Protein deubiquitination during oocyte maturation influences sperm function during fertilisation, antipolyspermy defense and embryo development. Reprod. Fertil. Dev..

[112] Bianchi E., Wright G.J. (2014). Izumo meets Juno: Preventing polyspermy in fertilization. Cell Cycle.

[113] Powrie E.A., Ciocanel V., Kreiling J.A., Gagnon J.A., Sandstede B., Mowry K.L. (2016). Using in vivo imaging to measure RNA mobility in Xenopus laevis oocytes. Methods.

[114] Li W., Wang Y.J., Zhu M., Fan T.T., Zhou D.M., Phillips B.L., Sparks D.L. (2013). Inhibition mechanisms of Zn precipitation on aluminum oxide by glyphosate: A 31P NMR and Zn EXAFS study. Environ. Sci. Technol..

[115] Hamad A.M. (2017). Molecular and Physical Interactions of Human Sperm with Female Tract Secretions. Ph.D. Thesis.

[116] Kerns K., Sharif M., Zigo M., Xu W., Hamilton L.E., Sutovsky M., Ellersieck M., Drobnis E.Z., Bovin N., Oko R. (2020). Sperm cohort-specific zinc signature acquisition and capacitation-induced zinc flux regulate sperm-oviduct and sperm-zona pellucida interactions. Int. J. Mol. Sci..

[117] Da Ros V.G., Muñoz M.W., Battistone M.A., Brukman N.G., Carvajal G., Curci L., Gómez-Elías M.D., Cohen D.J., Cuasnicu P.S. (2015). From the epididymis to the egg: Participation of CRISP proteins in mammalian fertilization. Asian J. Androl..

[118] Robertson S.A., Sharkey D.J. (2016). Seminal fluid and fertility in women. Fertil. Steril..

[119] Wang J.L., Zhang H.J., Wang H.L., Wang J.W., Gou P.H., Ye Z.H., Wang Y.L. (2015). Influence of hypothyroidism on oxidative stress, c-Fos expression, cell cycle, and apoptosis in rats testes. Toxicol. Environ. Chem..

[120] Palmer B.F. (2003). Sexual Dysfunction in Men and Women with Chronic Kidney Disease and end-stage kidney disease. Adv. Ren. Replace..

[121] Aarabi M., San Gabriel M.C., Chan D., Behan N.A., Caron M., Pastinen T., Bourque G., MacFarlane A.J., Zini A., Trasler J. (2015). High-dose folic acid supplementation alters the human sperm methylome and is influenced by the MTHFR C677T polymorphism. Hum. Mol. Genet..

[122] Zhu C., Lv H., Chen Z., Wang L., Wu X., Chen Z., Zhang W., Liang R., Jiang Z. (2017). Dietary zinc oxide modulates antioxidant capacity, small intestine development, and jejunal gene expression in weaned piglets. Biol. Trace Elem. Res..

[123] Wendlova J. (2013). Progression of the erectile dysfunction in the population and the possibilities of its regression with bioregeneration. Neuroendocrinol. Lett..

[124] Grieger J.A., Clifton V.L. (2015). A review of the impact of dietary intakes in human pregnancy on infant birthweight. Nutrients.

[125] Sengupta P. (2013). Environmental and occupational exposure of metals and their role in male reproductive functions. Drug Chem. Toxicol..

[126] Feki-Tounsi M., Hamza-Chaffai A. (2014). Cadmium as a possible cause of bladder cancer: A review of accumulated evidence. Environ. Sci. Pollut. Res..

[127] Lehtonen L., Gimeno A., Parra-Llorca A., Vento M. (2017). Early neonatal death: A challenge worldwide. Semin. Fetal Neonatal Med..

[128] Mridha M.K., Matias S.L., Chaparro C.M., Paul R.R., Hussain S., Vosti S.A., Harding K.L., Cummins J.R., Day L.T., Saha S.L. (2016). Lipid-based nutrient supplements for pregnant women reduce newborn stunting in a cluster-randomized controlled effectiveness trial in Bangladesh. Am. J. Clin. Nutr..

[129] Deshpande J.D., Joshi M.M., Giri P.A. (2013). Zinc: The trace element of major importance in human nutrition and health. Int. J. Med. Sci. Public Health.

[130] Mistry H.D., Kurlak L.O., Young S.D., Briley A.L., Broughton Pipkin F., Baker P.N., Poston L. (2014). Maternal selenium, copper and zinc concentrations in pregnancy associated with small-for-gestational-age infants. Matern. Child Nutr..

[131] Fabunmi T.M., Onabanjo O.O., Oguntona E.B., Keshinro O.O., Onabanjo J.A., Obanla O.O., Oyawoye O.O. (2013). Nutrient intakes and nutritional status of mothers and their under-five children in a rural community of Oyo state, Nigeria. Int. J. Child Health Nutr..

[132] Hooper L., Bunn D., Jimoh F.O., Fairweather-Tait S.J. (2014). Water-loss dehydration and aging. Mech. Ageing Dev..

[133] Jyotsna S., Amit A., Kumar A. (2015). Study of serum zinc in low birth weight neonates and its relation with maternal zinc. J. Clin. Diagn. Res..

[134] Darnton-Hill I., Mkparu U.C. (2015). Micronutrients in pregnancy in low-and middle-income countries. Nutrients.

[135] Tadi K.K., Alshanski I., Mervinetsky E., Marx G., Petrou P., Dimitrios K., Yitzchaik S. (2017). Oxytocin-monolayer-based impedimetric biosensor for zinc and copper ions. ACS Omega.

[136] Parkash A., Haider N., Khoso Z.A., Shaikh A.S. (2015). Frequency, causes and outcome of neonates with respiratory distress admitted to Neonatal Intensive Care Unit, National Institute of Child Health, Karachi. J. Pak. Med. Assoc..

[137] Nenkova G., Petrov L., Alexandrova A. (2017). Role of trace elements for oxidative status and quality of human sperm. Balk. Med. J..

[138] Qu F., Ying X., Guo W., Guo Q., Chen G., Liu Y., Ding Z. (2007). The role of Zn-α2 glycoprotein in sperm motility is mediated by changes in cyclic AMP. Reproduction.

[139] Saleh B.O.M. (2008). Status of zinc and copper concentrations in seminal plasma of male infertility and their correlation with various sperm parameters. Iraqi Acad. Sci. J..

[140] Colagar A.H., Marzony E.T., Chaichi M.J. (2009). Zinc levels in seminal plasma are associated with sperm quality in fertile and infertile men. Nutr. Res..

[141] Dissanayake D.M.A.B., Wijesinghe P.S., Ratnasooriya W.D., Wimalasena S. (2010). Relationship between seminal plasma zinc and semen quality in a subfertile population. J. Hum. Reprod. Sci..

[142] Khan M.S., Zaman S., Sajjad M., Shoaib M., Gilani G. (2011). Assessment of the level of trace element zinc in seminal plasma of males and evaluation of its role in male infertility. Int. J. Appl. Basic Med. Res..

[143] Hadwan M.H., Almashhedy L.A., Alsalman A.R.S. (2012). Oral zinc supplementation restore high molecular weight seminal zinc binding protein to normal value in Iraqi infertile men. BMC Urol..

[144] Sundaram V., Srinivas M., Gurunathan J., Rao K., Maniyan R.P., Balasundaram S. (2013). Influence of trace elements and their correlation with semen quality in fertile and infertile subjects. Turk. J. Med. Sci..

[145] Altaher Y.M., Abdrabo A.A. (2015). Levels of Zinc and Copper in seminal plasma of Sudanese infertile males. J. Adv. Med. Med. Res..

[146] Zhao J., Dong X., Hu X., Long Z., Wang L., Liu Q., Sun B., Wang Q., Wu Q., Li L. (2016). Zinc levels in seminal plasma and their correlation with male infertility: A systematic review and meta-analysis. Sci. Rep..

